# Unintentional weight loss, its associated burden, and perceived weight status in people with cancer

**DOI:** 10.1007/s00520-019-04797-y

**Published:** 2019-05-02

**Authors:** Eva Y. N. Yuen, Alexandra K. Zaleta, Shauna McManus, Joanne S. Buzaglo, Thomas W. LeBlanc, Kathryn Hamilton, Kevin Stein

**Affiliations:** 1 Cancer Support Community, Research and Training Institute, 520 Walnut Street, Suite 1170, Philadelphia, PA 19106 USA; 2 Vector Oncology AI, Concerto Health AI, 501 Boylston Street 10th Floor, Boston, MA 02116 USA; 3 Duke Cancer Institute, School of Medicine, Duke University, 2424 Erwin Road, Suite 602, Durham, NC 27705 USA; 4 Carol G Simon Cancer Center, Morristown Medical Center, 100 Madison Ave, Morristown, NJ 07960 USA

**Keywords:** Unintentional weight loss, Cancer, Perceived weight status, BMI weight status, Subjective experiences, Patient-reported outcomes

## Abstract

**Purpose:**

Unintentional weight loss (UWL) is a prevalent problem in people with cancer and is associated with poorer psychosocial outcomes. A gap exists in understanding whether and how perceived and/or weight status impacts experiences of UWL. Thus, we sought to examine subjective experiences of UWL in people with cancer, and whether *perceived* and/or actual weight status impacts these experiences.

**Methods:**

Participants were recruited through Cancer Support Community’s Cancer Experience Registry® and related networks. Participants completed an online survey that included the FAACT Anorexia-Cachexia subscale, and 19 items that captured six themes related to “beliefs and concerns” (positive beliefs, psychosocial impact, physical impact, cancer outcomes, self-esteem, relationships with others). Perceived weight status (PWS) was assessed using a single item. Body mass index (BMI) was calculated using self-reported weight and height measurements.

**Results:**

Of 326 respondents, 114 reported experiencing UWL. Over one-third misperceived their weight, with 29% perceiving weight status as below their BMI status. UWL in those with perceived weight status of overweight/obese was associated with positive beliefs. However, being underweight by BMI or perceiving oneself as underweight were both associated with greater concerns about weight loss. Perceived weight status of underweight compared to normal or overweight/obese weight status was associated with poorer psychosocial well-being, personal control, self-esteem, and relationships with others.

**Conclusion:**

In people with cancer, perceived weight status, rather than BMI, had greater impact on negative “beliefs and concerns” about UWL. Findings suggest assessment of both perceived and actual BMI to address the impact of UWL on psychosocial wellbeing.

Unintentional weight loss (UWL) is frequently reported in people with cancer, and while it can affect individuals across all cancer stages and types, it is most commonly experienced in lung, upper-gastrointestinal cancer, and advanced cancer populations [1]. The prevalence of cancer-related UWL has ranged from 31 to 87% depending on cancer type and stage [2–4]. The extent of UWL is influenced by a range of factors, including disease and treatment symptoms and side-effects that impair food intake and lead to malnutrition [2], as well as metabolic changes resulting from inflammation in response to the tumor or treatment [5–7].

UWL and muscle loss are strongly associated with impaired tolerance of cancer treatment, greater frequency of treatment interruption, longer hospital stays, and poor survival outcomes [8, 9]. In addition, UWL can result in physical and psychosocial sequelae [10, 11]. Poorer physical functioning has been reported in response to UWL in people with cancer [12]. Loss of perceived control, poor body image, avoidance of social interactions, and decreased health-related quality of life are also associated with UWL [13–15]. Further, in describing their lived experience, individuals have reported negative “beliefs and concerns” about UWL being an indicator of declining health, including closer proximity to end-of-life [16, 17].

While research on the subjective experiences of UWL in people with cancer has provided useful insights into the lived experiences of UWL, explorations have largely been conducted in the context of cancer cachexia, in which individuals experience clinically significant levels of weight loss [10]. In addition, these studies predominantly focus on people with advanced [10], head and neck [14], or lung [15] cancer. As a result, limited information is available on the subjective experience of unintentional weight loss in its early stages. Understanding the subjective experience of patients with early UWL for across the trajectory of cancer care, including their unique concerns, has the potential to guide the development of supportive resources and programs tailored to address patient needs and improve psychosocial outcomes.

Additionally, studies have shown associations between perceived weight status (PWS) and health-related quality of life (HRQOL) in both adult and adolescent general populations [18, 19]. PWS and actual BMI weight status can differ, with reports of up to 15 to 43% of individuals misperceiving their weight [18, 20–22]. However, findings on the associations between perceived weights on psychosocial health have been inconsistent. For example, some studies found that misperceptions of being under or overweight were associated with poorer health status, while misperceptions about normal weight (i.e., incorrectly perceiving oneself as normal weight, when they are underweight or overweight) were associated with better health status [21, 22]. By contrast, other studies found that under or overestimation of weight was associated with higher HRQOL [19, 22]. Given that UWL in people with cancer can lead to poorer prognosis and treatment outcomes, PWS has the potential to impact “beliefs and concerns” about one’s own health and disease trajectory. While some research has been done on this topic in general populations as outlined above, it is currently unclear in cancer patient populations whether and how PWS is associated with subjective experiences of UWL.

Thus, the current study aimed to characterize subjective experiences in response to UWL in a community sample of people with cancer. Specifically, we sought to examine whether PWS and actual body weight was associated with “beliefs and concerns” about UWL. Understanding the experiences of people with UWL, and in particular, whether and how PWS impacts these experiences, has the potential to guide development of tailored support and programs to promote psychosocial well-being in people with cancer.

## Methods

### Participant recruitment

Participants were recruited between August 2017 and February 2018 through Cancer Support Community’s (CSC) network of community-based affiliates/chapters, CSC’s Cancer Experience Registry®, and social media. Individuals aged 18 years or older with a diagnosis of cancer were eligible to participate. For the current study, the sample was limited to participants who reported UWL since their cancer diagnosis (*n* = 114). Participants provided informed consent prior to completing the online survey, which took approximately 30 min to complete. Ethics approval for the study was obtained from Ethical and Independent Review Services (E&I, Independence, MO; Study #15153).

### Measures

Demographic information (age, gender, race, ethnicity, education, employment status, and household income) and self-reported clinical information (cancer type, stage, treatment) were obtained via the online survey. Body mass index (BMI) of participants was classified according to World Health Organization (WHO) standards: underweight defined as < 18.5, normal weight as 18.5–24.9, overweight as 25.0–29.9, and obese as ≥ 30.0 [23–25]. Using self-reported weight (pounds) and height (feet, inches) measurements, BMI was calculated using Quetelet’s index (weight divided by height squared, multiplied by standard metric system conversion factor [703]) [26]. Percentage of weight lost was calculated using self-reported current weight compared to self-reported weight 6 months prior to cancer diagnosis.

PWS was assessed using a single item developed for the survey which asked participants about self-perception of their weight (How would you describe your current weight). The item had four-point response scale: underweight, normal weight, overweight, and obese.

“Beliefs and concerns” about appetite and weight loss were examined by using the Anorexia/Cachexia subscale (ACS) of the Functional Assessment of Anorexia/Cachexia Therapy (FAACT) scale [27, 28], and through 19 additional items developed by the research team. The ACS subscale is comprised of 12 items that assess anorexia/cachexia symptoms and patient concerns [27, 28]. The ACS subscale was designed for use in combination with the Functional Assessment of Cancer Therapy-General (FACT-G) to assess quality of life, or as a stand-alone measure. Items are rated with a 5-point Likert scale ranging from 0 (not-at-all) to 4 (very much) and summed (0–48), with lower scores indicating worse symptoms and concerns. Cronbach alpha in the current population was 0.73.

To examine subjective experiences about UWL, the research team developed 19 items related to positive and negative “beliefs and concerns.” The items were derived based on qualitative interviews with cancer survivors who experienced self-reported UWL, along with input from professionals with expertise in nutrition and weight, palliative medicine, and cancer. Three items captured positive beliefs about UWL (e.g., “I feel positive about my weight loss”). The remaining items captured negative “beliefs and concerns” across six themes: (1) psychosocial impact (three items; e.g., “My weight loss causes me to feel anxious or worried”), (2) physical impact (two items; e.g., “My weight loss causes me to feel physically weak”), (3) loss of control (two items; e.g., “My weight loss makes me feel like I have lost control over my nutrition and eating”), (4) weight loss on cancer outcomes (three items; e.g., “I believe that my weight loss is a sign that my cancer is getting worse”), (5) impact on self-esteem (*n* = 3; “My weight loss causes me to lose my sense of identity [who I am]”), and (6) weight loss on relationships with others (three items; “I try to hide the amount of weight I have lost from my family”). Participants indicated level of agreement using 5-point Likert scale, ranging from 0 (not-at-all) to 4 (extremely).

### Data analyses

Descriptive statistics were calculated for sociodemographic and clinical variables. PWS and BMI status were compared using Cohen’s kappa (*ĸ*). Contingency tables were used to examine whether participants’ PWS corresponded with BMI status. Pearson’s coefficient and independent sample *t* tests were used for continuous variables.

Analysis of variance (ANOVA) with pairwise post-hoc comparisons (Scheffé) was used examine the main and interaction effects of PWS and BMI status on ACS scores and “beliefs and concerns” items, and to examine main effects of PWS and BMI status on percentage of weight lost. Data analysis was conducted using SPSS Version 24.0 [29].

## Results

### Participant characteristics

Participant characteristics are described in Table 1. Of the 326 people with cancer who completed the survey, 114 reported experiencing UWL since diagnosis. Comparisons of these two groups showed no differences except with respect to general health ratings and those reporting that they were currently receiving chemotherapy (*p* < .05). Participants who had experienced UWL since diagnosis were predominantly female (80%), Non-Hispanic White (85%), with an average age of 59 years (SD = 11 years), and an average 5.8 years from their first cancer diagnosis (SD = 4.77 years). Commonly reported diagnoses included: breast (33%), blood (26%), prostate (10%), and ovarian (8%) cancer. Nearly one-third (29%) of participants reported being diagnosed with metastatic cancer.

**Table 1:** Descriptive characteristics of the sample

		Full patient sample (*n* = 326)		Patients with unintentional WL since diagnosis (*n* = 114)	
		*N*	%	*N*	%
Age		Mean = 58.56	SD (11.26)	Mean = 59.38	SD (10.89)
		Range 28–86 years		Range 32–86 years	
Gender identity	Female	254	78%	91	80%
	Male	71	22%	23	20%
Race	White	275	89%	98	89%
	African American	9	3%	2	2%
	Asian or South Asian	4	2%	1	1%
	Multiple races	10	3%	3	3%
	Other or Prefer not to share	11	4%	6	6%
Hispanic or Latino/a		18	6%	5	5%
Marital status	Single	48	16%	20	18%
	Married/partnered	212	68%	72	66%
	Separated or divorced	41	14%	15	4%
	Widowed	7	2%	2	2%
Education	No college	22	7%	10	9%
	Some college	50	16%	14	13%
	College degree	126	40%	40	36%
	Graduate or professional degree	111	36%	45	41%
	Prefer not to share	2	1%	1	1%
Annual income	< $20 K	24	8%	6	6%
	$20–39 K	40	13%	15	14%
	$40–59 K	49	16%	20	19%
	$60–79 K	30	10%	12	11%
	$80–99 K	36	12%	11	10%
	$100 K+	82	27%	24	22%
	Prefer not to share/I do not know	48	15%	20	19%
Employment	Full-time	90	29%	28	26%
	Part-time	54	18%	20	18%
	Retired	94	30%	33	30%
	Disability	52	17%	25	23%
	Unemployed	19	6%	3	3%
Cancer diagnosis	Breast	135	41%	38	33%
	Leukemia	30	9%	11	10%
	Prostate	26	8%	4	4%
	Lymphoma	21	6%	8	7%
	Multiple myeloma	20	6%	10	9%
	Lung	14	4%	7	6%
	Ovarian	12	4%	9	8%
	Colorectal	10	3%	5	4%
	Other	50	15%	19	17%
Years since diagnosis		Mean = 5.95	SD (4.97)	Mean = 5.80	SD (4.77)
		Range < 1–32 years		Range < 1–22 years	
Stage at diagnosis	0	9	3%	1	1%
	1	62	19%	17	15%
	2	66	20%	25	22%
	3	75	23%	28	24%
	4	54	16%	18	16%
	My cancer does not have a stage/NA	35	11%	17	15%
	Other/I do not know	24	7%	8	8%
Current staging	Localized	116	37%	38	35%
	Metastatic	58	19%	21	19%
	My cancer does not have a stage/NA	87	28%	30	27%
	I do not know	50	16%	21	19%
Current status	Diagnosed, but have never experienced a recurrence or relapse	50	16%	16	14%
	Currently experiencing cancer recurrence or relapse	72	22%	32	28%
	In remission or have no current evidence of disease	166	51%	52	46%
	Other/I do not know	37	12%	13	12%
Ever metastatic		78	24%	32	29%
Ever experienced recurrence		93	29%	37	33%
Treatment history	Current chemotherapy	54	23%	29	36%
	Current radiation therapy	9	4%	3	4%
	Current hormonal therapy	79	33%	18	24%
	Current oral therapy	47	21%	20	27%
	Current immunotherapy	22	10%	12	16%
	Ever received surgery	233	81%	76	80%
General health	Poor	6	2%	6	6%
	Fair	63	22%	24	24%
	Good	112	38%	45	45%
	Very good	87	30%	19	19%
	Excellent	25	9%	6	6%

### BMI status and PWS

Using WHO standards for BMI weight status, in the current sample, 9% were underweight, 51% normal weight, 20% overweight, and 21% obese (Table 2). Regarding PWS, 17% of respondents perceived themselves as underweight, 40% normal weight, 37% overweight, and 6% obese. Average BMI across the sample was 25.35 (SD = 5.77). Since few people perceived themselves as obese, we collapsed the overweight and obese categories for both perceived and actual weight, for analysis purposes.

**Table 2:** Height, weight, and weight classification

		Full patient sample (*n* = 326)		Patients with unintentional WL since diagnosis (*n* = 114)	
		*M*/*n*	SD/%	*M*/*n*	SD/%
Height (in feet)		5.46	0.29	5.43	0.28
		Range 4.75–6.25		Range 4.83–6.17	
Weight (in pounds)		168.27	42.61	153.29	37.37
		Range 91.00–370.00		Range 91.00–253.00	
Percentage of weight lost*		n/a		13.42	9.91
	s			Range 2–57%	
BMI		27.41	6.19	25.35	5.77
		Range 15.06–53.26		Range 15.06–41.88	
Perceived weight status	Underweight	23	7%	19	17%
	Normal Weight	105	32%	45	40%
	Overweight	153	47%	42	37%
	Obese	44	14%	7	6%
BMI weight status	Underweight	11	4%	9	9%
	Healthy weight	115	38%	53	51%
	Overweight	89	30%	21	20%
	Obese	86	29%	22	21%

*Calculated using self-reported current weight vs weight 6 months before diagnosis

### BMI status, perceived weight status, and percentage of weight lost

For percentage of weight lost using self-reported current weight, and weight 6 months prior to cancer diagnosis, ANOVA results showed no significant associations between BMI weight status and percentage of weight lost (F[2, 99] = 0.29, *p* = .75). No significant associations were found between percentage of weight lost and PWS (F[2106] = 2.52, *p* = .09).

### Anorexia and cachexia subscale scores

#### BMI weight status

For appetite and weight loss concerns using the ACS subscale, ANOVA results showed that BMI weight status was significantly associated with ACS scores (F[2102] = 3.94, *p* < .05); those with a BMI status of underweight were more likely to have lower ACS scores (indicating higher levels of appetite and weight concerns) compared to those with a BMI status of overweight/obese, but not compared to those with BMI normal weight.

#### PWS

For appetite and weight loss concerns, ANOVA results showed that weight perception was significantly associated with ACS scores (F[2112] = 11.25, *p* < .001). Participants who perceived themselves as underweight had lower ACS scores (indicating higher levels of appetite and weight concerns) than participants who perceived themselves as normal weight or overweight/obese.

### Subjective experiences about UWL: “beliefs and concerns”

Across the 19 items that explored “beliefs and concerns” about UWL, participants most strongly rated positive beliefs about UWL. Over one-third (34%) reported that they felt positive about their weight loss (Quite a bit to extremely), while 25% reported that their family was supportive of their weight loss, and 22% reported that their health care team was supportive of their weight loss (Table 3). The most strongly endorsed negative “beliefs and concerns” about UWL were related to the following themes: physical symptoms, lack of control, psychosocial outcomes, and impact on cancer outcomes. With respect to physical symptoms, over one-fifth (21%) reported that their weight loss made them feel physically weak (Quite a bit to very much), and 20% reported that their weight loss caused them to have problems with eating foods or not eating enough food. Regarding lack of control, 16% of the sample reported that their weight loss made them feel like they have lost control over their nutrition and eating. For psychosocial outcomes, 15% reported that their weight loss causes them to feel anxious or worried. Regarding impact on cancer outcomes, 14% reported that they believe their weight loss is a sign that their cancer is getting worse (Table 3).

**Table 3:** Weight loss (WL) “beliefs and concerns”

	*n*	Quite a bit to very much (%)	Somewhat (%)	A little bit to not at all (%)
Positive Beliefs				
I feel positive about my WL	112	34	16	50
My family is supportive of the weight that I have lost	112	25	24	51
My health care team is supportive of the weight that I have lost	111	22	29	50
Negative “beliefs and concerns”				
Physical symptoms				
My WL causes me to feel physically weak	112	22	12	67
My WL causes me to have problems with eating foods or not eating enough food	112	21	16	63
Psychosocial symptoms				
My WL causes me to feel anxious or worried	113	15	11	74
My WL causes me to feel frustrated	112	11	12	78
My WL causes me to feel sad or depressed	112	8	6	86
Loss of personal control				
My WL makes me feel like I have lost control over my nutrition and eating	113	15	12	73
My WL makes me feel like I have lost control over my health	112	13	14	73
Weight loss on cancer outcomes				
I believe that my WL is a sign that my cancer is getting worse	111	14	10	77
I believe that my WL will cause me to be unable to continue with my cancer treatments	112	7	4	89
I believe that my WL is a sign that I am approaching end of life or death	114	12	5	82
Impacts on self-esteem				
My WL causes me to lose my sense of identity (who I am)	114	5	6	89%
My WLs causes me to feel unproductive or useless	113	8	6	86%
My WL makes me feel like a burden to others	113	4	8	88%
Impacts on relationships with others				
I try to hide the amount of weight I have lost from my family	110	5	6	88%
My WL negatively affects my relationship with my family	113	1	6	93%
I try to hide the amount of weight I have lost from my health care team	113	1	3	97%

*n* = 114

#### BMI weight status

Using BMI weight status in ANOVA models with “beliefs and concerns” statements as outcome measures, differences in groups were found for one of 19 items only. Individuals with BMI normal weight were more likely to highly rate the items “My weight loss causes me to feel anxious or worried” compared to those who were overweight/obese (Table 4). No differences were found between underweight, and other BMI weight classifications.

**Table 4:** ANOVA models

	df	F	*p* value
BMI weight status			
My weight loss causes me to feel anxious or worried	(2101)	3.875	0.024
Perceived weight status			
My weight loss makes me feel like a burden to others	(2111)	4.722	0.011
My weight loss negatively affects my relationship with my family	(2111)	8.808	0.000
My weight loss makes me feel like I have lost control over my health	(2110)	4.908	0.009
My weight loss makes me feel like I have lost control over my nutrition and eating	(2111)	6.521	0.002
My weight loss causes me to lose my sense of identity (who I am)	(2110)	18.468	0.000
My weight loss causes me to feel sad or depressed	(2109)	6.121	0.003
My weight loss causes me to feel anxious or worried	(2109)	7.320	0.001
My weight loss causes me to feel frustrated	(2108)	10.683	0.000
I try to hide the amount of weight I have lost from my family	(2106)	5.330	0.006
My health care team is supportive of the weight that I have lost	(2107)	3.845	0.024
Misperception of weight status			
My weight loss negatively affects my relationship with my family	(2100)	4.87	0.010
I believe that my weight loss is a sign that I am approaching end of life or death	(2100)	3.49	0.034

Significant models only displayed

#### Perceived weight loss

By contrast, using PWS in ANOVA models, 10 of 19 “beliefs and concerns” items significantly differed across PWS. The items were related to the impact of UWL on five of six themes and are described below: positive outcomes (*n* = 1), psychosocial health (*n* = 3), personal control (*n* = 2), self-esteem (*n* = 2), and relationships (*n* = 2).

##### Positive outcomes of weight loss

One item related to positive outcomes of weight loss differed between PWS groups. Individuals who perceived themselves as overweight/obese had higher scores on the item, “My health care team is supportive of the weight that I have lost,” than those whose PWS was underweight (Table 4). No differences in ratings were found between those whose PWS was underweight and normal weight.

##### Psychosocial health

All three items related to the impact of weight loss on psychosocial health were scored more highly in individuals who perceived themselves as underweight compared to other weight categories. Participants who underestimated their weight had higher scores for both items, “My weight loss causes me to feel sad or depressed,” and “My weight loss causes me to feel frustrated” than those who perceived their weight as normal weight or overweight/obese (Table 4). For the item “My weight loss causes me to feel anxious or worried,” those who perceived themselves as either underweight or normal weight had higher scores compared to those who perceived themselves as overweight/obese. No differences in scores were found between those who perceived themselves as underweight, and normal weight (Table 4).

##### Loss of personal control

Both items related to UWL on loss of personal control (“My weight loss makes me feel like I have lost control over my health;” “My weight loss makes me feel like I have lost control over my nutrition and eating”) were scored more highly by individuals who perceived themselves as underweight, compared to those who perceived themselves as either normal weight, or overweight/obese (Table 4).

##### Self-esteem

Two items that examined impact of weight loss on self-esteem were scored more highly by those who perceived themselves as underweight compared to other weight categories. Individuals who perceived themselves as underweight had higher scores on the item “My weight loss causes me to lose my sense of identity (who I am)” than those whose perceived their weight as either normal weight, or overweight/obese (Table 4). In addition, those who perceived themselves as underweight had higher scores on the item “My weight loss makes me feel like a burden to others” than those whose perceived themselves as overweight/obese only (Table 4).

##### Impacts on relationships with others

Two items related to impact of weight loss on relationships with others had higher scores among individuals who perceived themselves as underweight compared to other weight categories. Participants who perceived themselves as underweight had higher scores for the item “My weight loss negatively affects my relationship with my family” compared to both self-reported normal weight and overweight/obese groups*.* In addition, those who perceived themselves as underweight had higher scores for the item “I try to hide the amount of weight I have lost from my family” than those whose perceived weight was overweight/obese only.

### Misperception of weight status

In examining discrepancies between PWS and BMI status, over one-third (39%) of participants misperceived their weight, with 29% whose PWS was below that of their BMI status (*ĸ* = − 0.192; *p* < .001; Table 5). We examined discrepancies between PWS and BMI status on ACS scores, and “beliefs and concerns” items using the following groups: perceived weight and BMI status match (correct weight status); PWS above BMI status (overestimated), PWS below BMI status (underestimated). No differences were found between groups on ACS scores.

**Table 5:** Objective and subjective weight classification comparison

	How would you describe your current weight?					
BMI-based weight classification		Underweight	Normal weight	Overweight	Obese	Total
	Underweight	8	1	0	0	9
	Normal Weight	9	34	9	0	52
	Overweight	0	5	16	0	21
	Obese	0	0	16	6	22
	Total	17	40	41	6	104

*ĸ* = − 0.192; *p* < .001

Differences between groups were found for two “beliefs and concerns” items. Those who underestimated their weight had higher scores for the item “My weight loss negatively affects my relationship with my family,” compared to those who correctly self-reported their weight (Table 4). Further, those who underestimated their weight more strongly endorsed the item “I believe that my weight loss is a sign that I am approaching end of life or death” compared to those who overestimated their weight (Table 4).

## Discussion

While studies have examined the subjective experiences of considerable weight loss in people with cancer, the current findings suggest that even non-clinically significant weight loss can be a source of concern. Distinct from other studies, we found that PWS, rather than BMI status itself, had greater impact on negative “beliefs and concerns” about UWL in a community sample of people with cancer. Specifically, the perception of being underweight was associated with more adverse “beliefs and concerns” about appetite and weight loss compared to the perception of being normal weight or overweight or obese. Notably, these adverse “beliefs and concerns” about *perceived* weight loss were similar to concerns identified in people with clinically significant levels of weight loss [30]. To our knowledge, this is a novel finding in patients with cancer.

Over one-third of participants (39%) in our study misperceived their weight, with 29% who underestimated their weight, compared to their BMI status, a finding similar to existing studies in general populations [31–33]. In support of existing studies [30], we found that UWL was associated with negative “beliefs and concerns”, including lack of control, distress and anxiety around weight loss, and disease progression and proximity to end-of-life or death. Importantly, in our study, a perceived status of underweight, regardless of BMI grouping, was associated more adverse psychosocial outcomes, including feeling anxious, worried, or frustrated with weight loss, as well as concerns about loss of control over their health, nutrition, and eating. People who perceived themselves as underweight also had poorer psychosocial outcomes and reported negative impact of weight loss on relationships with their family and healthcare clinicians. Individuals who underestimated their weight were also more likely to view weight loss as a sign they were approaching end-of-life Together, these findings suggest that an individual’s PWS, rather than their actual BMI, plays a greater role in shaping their “beliefs and concerns” about the consequences of UWL. Our findings highlight that assessment of individuals ought to include changes in PWS as well as actual BMI, to address both objective and subjective factors that influence psychosocial wellbeing. Notably, individuals with a normal-range BMI were more likely to report feeling anxious or worried about their weight loss, and that their weight loss made them feel physically weak, compared to those with a BMI status of overweight/obese. These findings further suggest that a BMI status of healthy weight alone may inaccurately predict an individual’s beliefs about their weight loss in response to cancer. Indeed, weight loss has been identified as a possible risk factor for poorer health-related quality of life, and poorer prognosis [13], and as such, weight loss across all BMI status’ could be considered as an indicator for the need for further assessment and intervention to address impacts on an individual’s psychosocial wellbeing. Further, research has shown that oncology clinicians are reluctant to discuss the topic of weight management in cancer survivorship [34]. However, the current findings highlight the importance of weight management discussions with people with cancer across all weight categories, and in particular, those of normal weight with UWL, given that objective measures (BMI status) of weight alone are not a sufficient assessment of a patient’s experience of UWL. Not surprisingly, those with a PWS of overweight or obese were more likely to report that their healthcare team was supportive of their weight loss. In cancer survivors, excess weight has been identified as a risk factor for recurrence, second primary cancers, reduced treatment efficacy, increased treatment complications, and poorer survival [35–38]. Thus, for people who are overweight/obese, reductions in weight may have been reviewed in a positive light by healthcare clinicians and may even be reinforced. Indeed, beliefs about health benefits of weight loss in overweight people has been reported [16], including improved body image [39, 40]. However, sarcopenia has been found in overweight/obese people with cancer [41], suggesting weight loss should be monitored for people with cancer across all weight classifications.

Notably, a small proportion (10%) of the sample overestimated their weight compared to their BMI status; these individuals were either normal weight (*n* = 9) or underweight (*n* = 1). Studies have shown that overestimation of weight for those with normal weight, compared to accurate perception, has been associated with depressive symptoms in women, but not men [42, 43]. Although outside the scope of the study, for those with a history of eating disorders, bariatric surgery, body dysmorphia, and/or long-term difficulty managing excess weight may view the UWL favorably, and continue to perceive themselves as overweight, when their BMI status suggests otherwise. Further research is recommended to examine impacts of perceived overestimation of weight following UWL in people with cancer.

Although the ACS subscale was designed to assess appetite and weight concerns in people experiencing considerable weight loss, the current study suggests that it may also be useful in identifying concerns in people who have experienced non-clinically significant levels of UWL following a cancer diagnosis. Further studies could assess the utility of the ACS subscale in larger cancer populations.

### Limitations and future research

A limitation of the study was the reliance on self-reported weight and height measurements to determine BMI classifications, which has the potential for misclassification of participants if their reports were inaccurate. In addition, the study was descriptive, had a sample comprised of predominantly White, educated, and female participants, and lacked a comparison group. Recommendations for future studies include objectively derived height and weight measurements to calculate BMI, as well as comparisons of “beliefs and concerns” among individuals with and without UWL. We also recommend further examination of how PWS, BMI status, and misperceptions about weight status influences “beliefs and concerns” about UWL, using longitudinal or causal design studies. We also recommend examining associations between PWL, “beliefs and concerns” about UWL, and clinical factors such as time since diagnosis, disease stage and status to better understand impacts of UWL on adjustment to illness and perceived risk of impairment or demise. Understanding the impact of appetite loss on PWL and “beliefs and concerns” about UWL and its associations with misperceptions about cancer, and adverse psychosocial outcomes is also recommended. Further, implications for the development and evaluation of clinical interventions may include methods that foster greater patient engagement and collection of patient-reported outcomes around perceptions and concerns around weight loss with alerts to the clinical team for referral and follow-up.

## Conclusions

The study provides insight into the subjective experiences of UWL in people with cancer, and highlights the impact of PWS, rather than BMI status, on negative “beliefs and concerns” about UWL. Our study shows that even non-clinically significant weight loss can affect psychosocial wellbeing of people with cancer, and that perceptions about weight loss are just as important as the reality. Results from the current study constitute the first step in understanding the impact of PWS on the “beliefs and concerns” about UWL in people with cancer. These findings provide a useful starting point for healthcare clinicians, educators, and researchers to identify specific concerns about UWL. Further understanding of the subjective experiences, and *PWS* in response to UWL can inform efforts to develop, evaluate and implement community-based supportive interventions and self-management programs to enhance psychosocial well-being in people experiencing UWL, and promote effective communication with their healthcare team about their UWL concerns.
