# Correction to: Unintentional weight loss, its associated burden, and perceived weight status in people with cancer

**DOI:** 10.1007/s00520-022-07202-3

**Published:** 2022-06-09

**Authors:** Eva Y. N. Yuen, Alexandra K. Zaleta, Shauna McManus, Joanne S. Buzaglo, Thomas W. LeBlanc, Kathryn Hamilton, Kevin Stein

**Affiliations:** 1 Cancer Support Community, Research and Training Institute, 520 Walnut Street, Suite 1170, Philadelphia, PA 19106 USA; 2 Vector Oncology AI, Concerto Health AI, 501 Boylston Street 10th Floor, Boston, MA 02116 USA; 3 Duke Cancer Institute, School of Medicine, Duke University, 2424 Erwin Road, Suite 602, Durham, NC 27705 USA; 4 Carol G Simon Cancer Center, Morristown Medical Center, 100 Madison Ave, Morristown, NJ 07960 USA

**Correction to: Supportive Care in Cancer (2020) 28:329–339**

10.1007/s00520-019-04797-y

The article “Unintentional weight loss, its associated burden, and perceived weight status in people with cancer”, written by Yuen, E.Y.N, Zaleta, A.K., McManus, S., Buzaglo, J.S., LeBlanc, T.W., Hamilton, K., Stein, K., was originally published Online First without Open Access. After publication in volume 28, issue 1, page 329–339 the author decided to opt for Open Choice and to make the article an Open Access publication. Therefore, the copyright of the article has been changed to © The Author(s) 2022 and the article is forthwith distributed under the terms of the Creative Commons Attribution 4.0 International License, which permits use, sharing, adaptation, distribution and reproduction in any medium or format, as long as you give appropriate credit to the original author(s) and the source, provide a link to the Creative Commons licence, and indicate if changes were made. The images or other third party material in this article are included in the article’s Creative Commons licence, unless indicated otherwise in a credit line to the material. If material is not included in the article’s Creative Commons licence and your intended use is not permitted by statutory regulation or exceeds the permitted use, you will need to obtain permission directly from the copyright holder. To view a copy of this licence, visit http://creativecommons.org/licenses/by/4.0.

The original article is corrected.

